# Semi-automated quantification of living cells with internalized nanostructures

**DOI:** 10.1186/s12951-015-0153-x

**Published:** 2016-01-15

**Authors:** Michael Bogdan Margineanu, Khachatur Julfakyan, Christoph Sommer, Jose Efrain Perez, Maria Fernanda Contreras, Niveen Khashab, Jürgen Kosel, Timothy Ravasi

**Affiliations:** Division of Biological and Environmental Sciences and Engineering, KAUST Environmental Epigenetic Program (KEEP), King Abdullah University of Science and Technology (KAUST), Thuwal, 23955 Kingdom of Saudi Arabia; Division of Computer, Electrical and Mathematical Sciences and Engineering, King Abdullah University of Science and Technology, Thuwal, Kingdom of Saudi Arabia; Division of Physical Science and Engineering, Smart Hybrid Materials Laboratory (SHMs), King Abdullah University of Science and Technology,, Thuwal, Kingdom of Saudi Arabia; Institute of Molecular Biotechnology of the Austrian Academy of Sciences (IMBA), Dr. Bohr-Gasse 3, Vienna, 1030 Austria

**Keywords:** Nanomedicine, Nanoparticle, NWs, Magnetic, Quantification, Live-cell imaging, Machine learning, Computational methods

## Abstract

**Background:**

Nanostructures fabricated by different methods have become increasingly important for various applications in biology and medicine, such as agents for medical imaging or cancer therapy. In order to understand their interaction with living cells and their internalization kinetics, several attempts have been made in tagging them. Although methods have been developed to measure the number of nanostructures internalized by the cells, there are only few approaches aimed to measure the number of cells that internalize the nanostructures, and they are usually limited to fixed-cell studies. Flow cytometry can be used for live-cell assays on large populations of cells, however it is a single time point measurement, and does not include any information about cell morphology. To date many of the observations made on internalization events are limited to few time points and cells.

**Results:**

In this study, we present a method for quantifying cells with internalized magnetic nanowires (NWs). A machine learning-based computational framework, CellCognition, is adapted and used to classify cells with internalized and no internalized NWs, labeled with the fluorogenic pH-dependent dye pHrodo™ Red, and subsequently to determine the percentage of cells with internalized NWs at different time points. In a “proof-of-concept”, we performed a study on human colon carcinoma HCT 116 cells and human epithelial cervical cancer HeLa cells interacting with iron (Fe) and nickel (Ni) NWs.

**Conclusions:**

This study reports a novel method for the quantification of cells that internalize a specific type of nanostructures. This approach is suitable for high-throughput and real-time data analysis and has the potential to be used to study the interaction of different types of nanostructures in live-cell assays.

**Electronic supplementary material:**

The online version of this article (doi:10.1186/s12951-015-0153-x) contains supplementary material, which is available to authorized users.

## Background

The field of nanoparticles for biomedical applications has drawn increasing attention, especially because of their small size, which allows them to penetrate and interact with a single cell and its intracellular components [1–6]. Magnetic nanoparticles have shown specific advantages due to the remote control by magnetic fields and have been employed as tools to tackle challenges in biology and medicine [7–13].

Nanostructures such as NWs and nanotubes are nowadays a powerful tool for cellular delivery and sensing [14]. An interesting feature of magnetic NWs is that their diameter and length can be independently modulated [15, 16]. They have a larger magnetic moment per unit of volume compared to beads [17] and, due to their shape anisotropy, they can have permanent magnetic properties [18], enabling the exertion of torques. Such torques have recently been exploited for killing cancer cells by stimulating NWs with a low-frequency magnetic field resulting in oscillations that caused cells’ apoptosis [19]. NWs can exert a death-inducing effect on cancer cells without being internalized but only in contact with the cell membrane, whereby they can induce mechanical stress [19]. Similar results were found by Kim et al. [20], who induced cancer cells death with vibrating magnetic microdisks. Nevertheless, in order to improve the therapeutic efficiency of nanostructures in biomedical applications, a proper understanding of their uptake by cells and internalization kinetics is needed. This will help in designing nanoparticles that can easily enter the cells and in understanding both their adverse and favorable aspects [21].

Several approaches have been used to functionalize nanostructures for various applications at cellular level [22, 23], and quantify the internalization of these nanostructures by different cell lines [24–31]. Most of the internalization studies with NWs and other nanoparticle types conducted so far have limited time resolution; in particular for cellular uptake, the quantification is most of the time based on visual approximations by the experimentalist and thus not suitable for high-throughput analysis and large data sets [21, 32–37]. However, high-throughput studies have previously addressed quantification of the number of particles internalized by cells and thus indirectly also provided a quantification of the number of cells with and without any particles [38–41]. Even though these approaches are able of analyzing large numbers of cells, these approaches are nevertheless limited to single time point measurements and requiring additional preparation steps.

Microscopy approaches are qualitative and limited to small populations of cells, sometimes even single cells. It is however possible to generate extensive microscopy time-lapse acquisition data; in this case then the bottleneck resides at quantitative image analysis, and this is one of the challenges to which the current study aims to respond. Although the automated analysis is feasible, most of the time evaluation of experimental results is performed manually. Many of the supervised and unsupervised machine learning methods have great potential for quantitative and semi-quantitative nanoparticle uptake investigations, however in the literature there is very limited number of studies which take advantage of these high-end computational tools [42].

A highly innovative method that integrates high-resolution confocal microscopy with automatic image analysis was previously reported [43]. The method is called Particle_in_Cell-3D and was applied to precisely quantify the cellular uptake of silica and ceria nanoparticles [43]. It can determine the position and intensity of all particles, the number of intracellular particles and membrane-associated particles, as well as the concentration of particles [43]. The focus isn’t however on quantifying the number of cells with internalized particles.

The goal of this study was to develop a semi-automatized method for quantification of cells which uptake NWs, method that moves away from conclusions solely drawn on visual observations made by experimentalist, and can complement flow cytometry-based techniques. The new method is based on a fast computational framework initially developed as a tool to investigate cell division and dedicated to time-resolved analysis of single cells, CellCognition.

There is a large variety in the types of NWs available for research, their surface functionalization, and at the same time the diversity of cell lines, incubation conditions, and doses used in experiments. With this strategy, comparable cellular uptake studies can be conducted to investigate how the unique properties of NWs influence their internalization, and better assess their effects on the respective cell lines [44].

In order to verify the developed method, internalization studies were conducted with Fe and Ni NWs. Both NWs have a thin oxide layer as their outer shell, which was utilized for coating them with (3-aminopropyl) triethoxysilane (APTES), and subsequently labeling them with a fluorogenic pH-dependent dye pHrodo™ Red. Live-cell time-lapse imaging studies were conducted for 24 h (h) for the Fe and Ni NWs with two different cell lines, HCT 116 and HeLa.

## Results and discussion

### The cellular uptake quantification pipeline

The pipeline reported in this study consists of three main components: coating of nanoparticles with APTES and labeling with fluorogenic pH-dependent dye pHrodo™ Red, live-cell time-lapse imaging studies, and image analysis using a machine learning based computational framework.

The choice of using pHrodo™ Red dye was due to its distinctive fluorescence inside and outside the cell. pHrodo™ Red has been previously used successfully by Arppe et al. for studying cellular uptake of UCNP (upconversion nanoparticle) probes [37]. The method chosen for visualizing the interaction was fluorescence microscopy, as it can be performed on live cells with high spatial and temporal resolution. A computational framework dedicated to automatic analysis of live cell imaging data, CellCognition, has been adapted and used to classify the cells with internalized and non-internalized NWs, based on the pHrodo™ Red-characteristic fluorescence, and subsequently determine the uptake percentage by cells at different time points.

### NWs characterization

The surface charges, zeta potential, of the APTES-coated Fe NWs was −17.4 mV whereas that of non-coated Fe NWs was 2.85 mV. For Ni NWs, the **ζ** potential of the APTES-coated Ni NWs was −14 mV. The negative value obtained for the APTES-coated NWs is confirmed by the study of Arppe et al. [37], in which the zeta potential of the amino-silane coated UCNPs before conjugation was measured as −22 mV at pH 7.2 [37]. This could be explained by the high abundance of acidic silanols (Si–OH) on the surface of NWs [37].

**Fig. 1:**
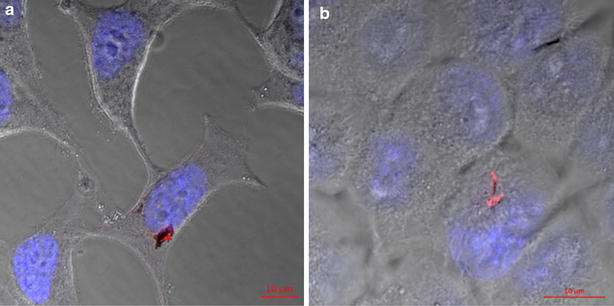
**a** HeLa cells incubated for 18 h with pHrodo™Red-tagged Fe NWs. **b** HCT 116 cells incubated for 6 h with pHrodo™Red-tagged Ni NWs. In blue-nuclei stained with Hoechst 33342. In red the pHrodo™Red characteristic signal of internalized Fe NWs

The FTIR (Fourier transform infrared spectroscopy) characterization of non-coated and APTES-coated NWs made of Fe and Ni are shown in the Additional files 1 and 2 respectively. A prominent peak can be observed for both the APTES-coated Fe NWs and the APTES-coated Ni NWs at a wavenumber of approximately 1000 cm^−1^. The peak corresponding to Si–O is of approximately 1054 cm^−1^ according to experimental data found in the literature [45]. These results along with the confocal laser scanning microscopy images of cells incubated with NWs shown in Fig. 1, provide a direct confirmation of the initial coating step with APTES and successful tagging with pHrodo™ Red.

TEM images of single APTES-coated Fe NW and Ni NW are shown in Additional files 3, 4. The TEM images are showing the dimensions of a single nanowire and the oxide layer thickness.

### Cellular uptake studies with Fe and Ni NWs

In this study “proof-of-principle” time-lapse imaging experiments for 24 h were conducted with two different cell lines and two NW materials.

The human epithelial colorectal carcinoma cell line HCT 116 was chosen for cytotoxicity studies. The cell line comes from colon epithelial tissue, making it a good cytotoxicity model, as epithelium represents a common exposure tissue for biomaterials [19].

Another cell line that was used in this project is HeLa. The HeLa cell line has low cellular motility; it is relatively insensitive to light exposure and has been used in previous internalization studies with different nanoparticles types [43, 46].

Both Fe and Ni NWs have been used for comparative purposes. NWs were added to the cells at ratios of approximately 200 NWs per cell, based on the cytotoxicity studies conducted by Perez et al. [47]. From all investigated ratios, the 200:1 was the largest suitable for killing HCT 116 cancer cells, while not inducing severe cytotoxicity in normal somatic cells [47]. The same ratio has also been used for HeLa.

Two time-lapse videos, one showing HeLa cells and pHrodo™ Red-tagged Ni NWs (Additional file 5), and the other HeLa cells and pHrodo™ Red-tagged Fe NWs (Additional file 6) allow us to observe that cells tear large NW aggregates down, and that cells can divide with NW aggregates inside.

A control experiment without cells was run with Fe NWs in cell imaging medium with a pH of 8.2 and no fluorescence was observed (Additional file 7). However, the NWs do fluoresce brightly at pH 6.2, which corresponds to the pH value inside early endosomes [48].

For the secondary pHrodo™ Red channel, the object counts for each of the two classes—“nanonegative” and “nanopositive”—were calculated by the software. The number of “nanopositive” cells was further divided by the total number of cells to calculate the percentage of “nanopositive” cells, which was accordingly plotted at each time point of the image acquisition series.

**Fig. 2:**
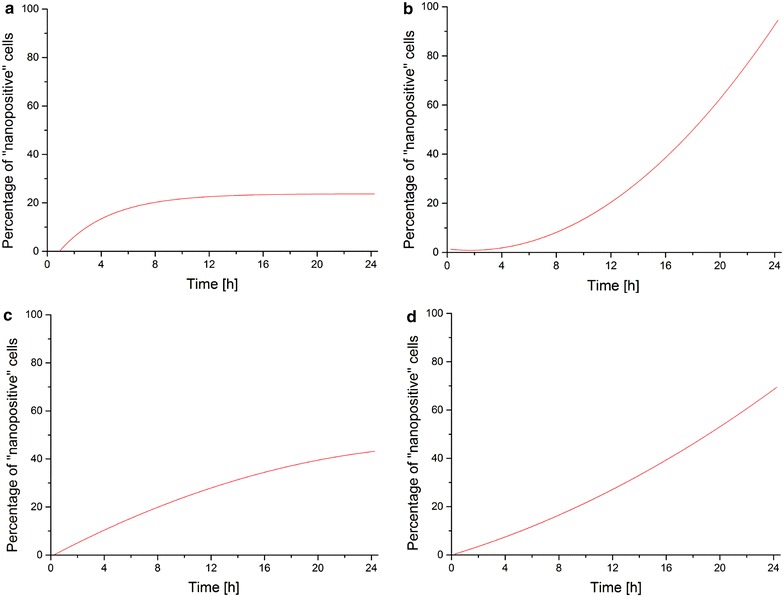
Proof-of-concept cellular uptake studies with Fe and Ni NWs. Regression fit curves for mean percentage values of “nanopositive” cells with internalized nanowires were plotted across time. In each condition, the averaging was done for three different areas in a cell culture dish, each having a distinct population of cells (numbering 500–600 at the end of the time-lapse experiment). **a** “Nanopositive” HeLa cells with internalized Fe NWs. **b** “Nanopositive” HCT 116 cells with internalized Fe NWs. **c** “Nanopositive” HeLa cells with internalized Ni NWs. **d** “Nanopositive” HCT 116 cells with internalized Ni NWs

The regression fit curves for the average “nanopositive” percentage values calculated for three independent positions located in the same cell culture dish at each time point of the 24 h time-lapse were plotted in Fig. 2. The fit curves allow for observations on nanowires internalization patterns, however it should be mentioned that based on these observations the aim is not to draw any conclusions related to the internalization pathway, cell viability or cytotoxicity as it is not the focus of our study.

There is variability among the three positions investigated within the same dish used for the experiment. This could be explained by the nonspecific distribution of both Fe NW and Ni NW structures, with multiple aggregates of different sizes. Therefore there is no homogenous consistent and comparable NWs distribution even for independent positions within the same dish. It should be also noted that the cell cycle was not synchronized, and therefore there are differences in the proliferation of cells captured at various locations within the cell culture dish.

For HeLa cells incubated with Fe NWs the mean percentage of “nanopositive” cells after 24 h post-incubation with NWs is approximately 20 ± 0.86 %, and over the 24 h time interval, the cellular uptake shows a good fit to an asymptotic function (R^2^ = 0.949), as shown in Fig. 2a.

The relatively high proliferation of HeLa cells in this experiment (increase of approximately 60 % in the number of total cells after 24 h), could explain this logarithmic “behavior.” The logarithmic-style increase gives the impression that there will be no additional cells internalizing NWs between 8 and 24 h post-incubation; however, this is not the case. With the new cells resulting from proliferation, cells which would be initially classified as “nanonegative”, it would be expected to see a drop in the percentage of “nanopositive” cells. That phenomenon cannot be observed, thus providing an indirect indication that during cell doubling additional cells from the population internalize NWs, and thus the percentage of “nanopositive” cells is maintained relatively constant.

Figure 2b shows that, for HCT 116 cells incubated with Fe NWs, the mean percentage of “nanopositive” cells is approximately 89 ± 2.3 % after 24 h. It can be observed that the internalization process is slower at the beginning, given the low percentage values at the early time points. It is also possible to get an indication that at the later time points there are cells that just start to internalize nanowires and their number is large.

For HeLa cells incubated with Ni NWs, the CecogAnalyzer results were plotted in Fig. 2c. The mean percentage of “nanopositive” cells after 24 h post-incubation with NWs is approximately 45 ± 11.9 %, and over the 24 h time interval, the cellular uptake shows very good fit to a polynomial function (R^2^ = 0.988) (Fig. 2c). In this case, the number of total cells increased by 15 % after 24 time lapse (data not shown). For the first 8 h, there seems to be a linear fit aspect, however after 8 h, the function looks similar to the asymptotic function observed in the case of HeLa and Fe NWs. Compared to the case of HeLa and Fe NWs, internalization at later time points seems to be more effective in the case of the Ni NWs.

In the case of HCT cells and Ni NWs, the mean percentage of “nanopositive” cells is approximately 58 ± 6.96 % after 24 h, and over the 24 h time interval, the cellular uptake shows very good fit to a polynomial function (R^2^ = 0.991), as shown in Fig. 2d. The internalization of Ni NWs by HCT 116 cells follows a steady increase, which also seems to show a good linear fit. At the beginning of the time lapse (first 6 h), there are more HCT 116 cells with first internalization events, in terms of percentage values, with Ni NWs as compared with Fe NWs.

Some differences can be noted in the uptake behavior of the two cell lines. For both Fe and Ni NWs the cellular uptake recorded with HCT 116 cells was higher than the cellular uptake of HeLa cells. The lower percentages obtained for HeLa cells could be explained by previous findings [43, 46]. In the study of Torrano et al. [43], it was shown that human vascular endothelial cells (HUVEC) are more efficient in incorporating particles within the first 4 h of incubation, with the number of intracellular particles, up to 10 times higher than for HeLa cells derived from the cervix carcinoma. HeLa cells are reported by dos Santos et al. [46] to show the highest proportion of cells with no particles, indicating their inability to internalize 1 μm-sized particles With the 1 μm-diameter particles, the probability of having cells with no particles internalized after the given exposure time was 74 % for HeLa. This corresponds to our observations with HeLa cells.

A comparison with full manual annotation was conducted for two different time points of three independent positions for the experiment with HCT 116 cells and Fe NWs. For the time point corresponding to 12 h post-incubation, approximately 91 % of the “nanopositive” cells counted by the software were also annotated as “nanopositive” by the experimentalist (average calculated across all three positions), while in the case of the last time point of the experiment (corresponding to 24 h post-incubation), there was agreement with approximately 99 % of the “nanopositive” cells counted by the software. The manual validation confirms that the semi-automated method is reliable and can be used with confidence. For the earlier time point, the experimentalist’s manual count was lower than the automated software count. However, the differences in pHrodo™ Red signal brightness cannot be easily distinguished by eye and the average pHrodo™ Red signal intensity is lower at the earlier time point as compared to the last time point investigated.

**Fig. 3:**
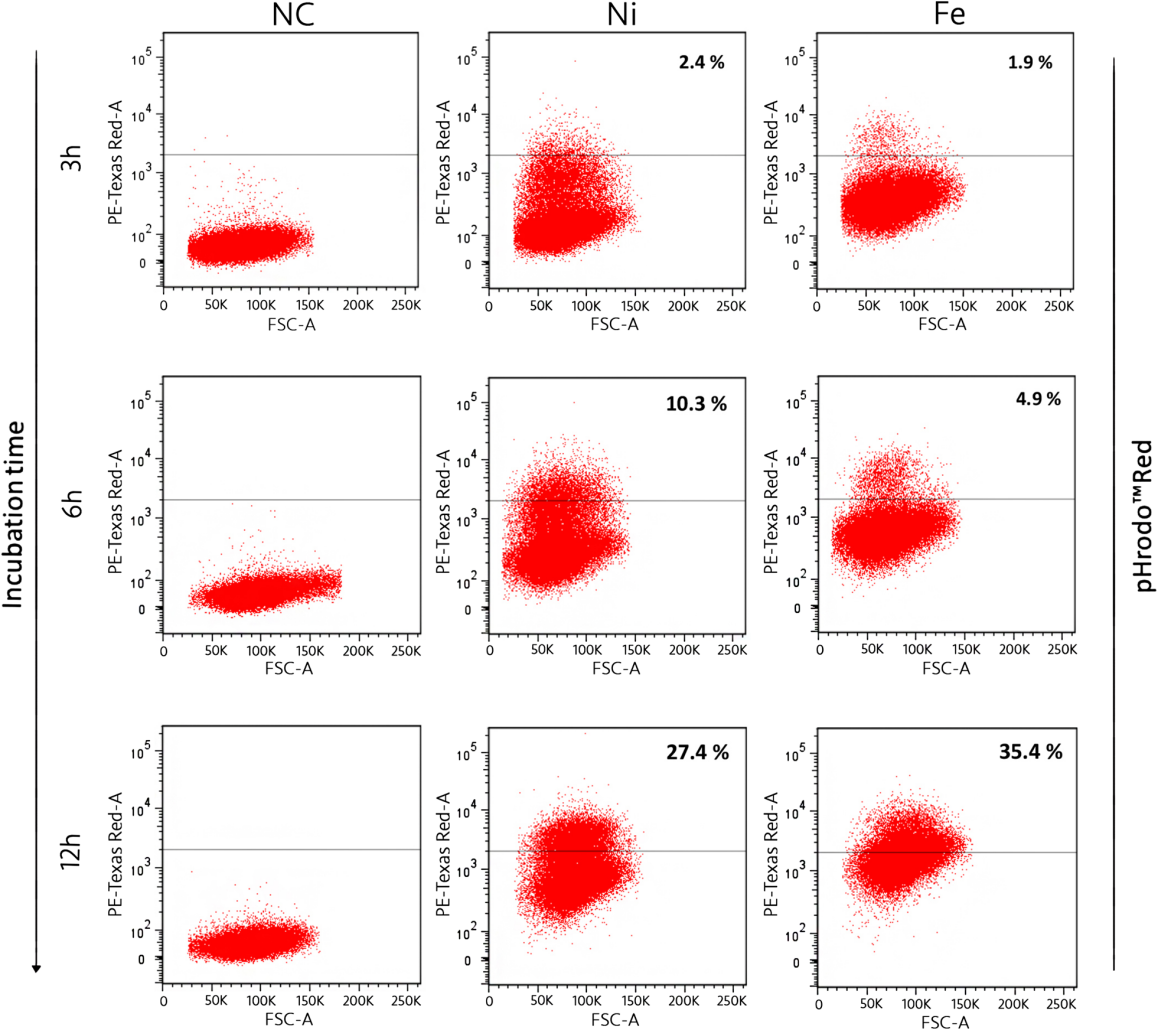
Fluorescence-activated cell sorting analysis of Fe and Ni NWs uptake by HCT 116 cells. Plots show the pHrodo™Red fluorescence intensities of HCT 116 cell populations after 3, 6 and 12 h incubation with Fe NWs and Ni NWs respectively. Numbers in quadrants indicate percentage of total cells with signal above threshold determined based on non-specific signal intensities from negative control (NC), representing HCT 116 cells incubated without NWs addition

Given the high throughput strength of flow cytometry and the advantages of pHrodo™ Red sensitivity, fluorescence activated cell sorting (FACS) has been used to validate the methodology presented in this study. While our observations with CellCognition were based on groups of 500–600 cells, for FACS the percentage values of “nanopositive” and “nanonegative” cells were based on up to 50000 cells. Cell sorting and analysis was performed on HCT116 cells incubated with Fe and Ni NWs respectively (Fig. 3). The obtained values for the condition with Ni NWs (2.4 % at 3 h, 10.3 % at 6 h, 27.4 % at 12 h) matched well the values obtained with CellCognition software (5.5 ± 3.31 % at 3 h, 8.1 ± 3.6 % at 6 h, 27.8 ± 1.50 % at 12 h). In the case of HCT 116 cells and Fe NWs FACS results indicated 1.9 % at 3 h, 4.9 % at 6 h, and 35.4 % at 12 h. The corresponding percentage values from CellCognition analysis were 2.36 % ± 1.30 % at 3 h, 4.21 ± 0.28 % at 6 h, and 19.72 ± 1.49 % at 12 h. The higher percentage value obtained with FACS for HCT 116 cells and Fe NWs might be due to low fluorescence signal on the outer surface of the cell membrane, caused by interaction of the dye with lysine residues, signal which however is not captured by our CellCognition analysis given the manual annotation but could be picked up by FACS. Limitations of the dye in this regard have been previously reported [27]. Differences are expected considering the number of cells analyzed with CellCognition compared to the high throughput of FACS. Nevertheless, additional frames and cell populations can be used for the CellCognition analysis; in parallel, multiple areas with different cell populations can be analyzed.

## Conclusions

This study reports the utilization of a machine learning-based method that is compatible with live cell-imaging experiments, suitable for semi-automated quantification of the number of cells with internalized nanostructures and useful for investigating the time dependent behavior of internalization, for the first time.

The method developed in this study has been tested for investigating the uptake “behavior” for Fe and Ni NWs in two different cell lines, HCT 116 and HeLa. The preliminary data indicate that over a 24 h time interval, both Fe and Ni NWs better target HCT 116 cells, as compared to HeLa cells.

Overall, this pipeline allows for comparison studies with different types of nanostructures (varying size, shape, and surface properties) across multiple cell lines. With specifically targeted improvements, the present study can set the basis for a new approach targeted at investigating cell-nanostructures interactions with the aid of machine learning, which would ideally be faster and more cost-effective than currently available methods based on microscopy imaging. Such a tool could be used with ease for large imaging data sets, thus reducing the workload for the experimentalist, and ensuring high-level consistency analysis. While lacking the high throughput power of FACS, the method presented in our study can complement flow cytometry technique with enhanced time resolution and cell morphology information.

## Methods

### NWs fabrication

The fabrication of Fe and Ni NWs was performed using the electrochemical deposition method on nanoporous alumina templates. A highly ordered porous aluminum oxide (PAO) membrane was prepared by the two-step anodization technique; the pores were then filled with Fe or Ni respectively, using pulsed electrodeposition, resulting in NWs with a perpendicular orientation with respect to the membrane surface and a length distribution dependent on the deposition time [49, 50]. The fabrication protocol with the different steps followed is described in detail elsewhere [49, 50].

The length of Fe NWs was approximately 3.5 μm, and that of Ni NWs was approximately 4.5 μm. The diameter of both Fe and Ni NWs was 35 nm.

### Coating of NWs with APTES

Ethanol absolute, sodium hydroxide (NaOH), and (3-aminopropyl) triethoxysilane (APTES) (≥98 %) were purchased from Sigma-Aldrich.

NWs (0.225 mg) were dissolved in ethanol, absolute, (≥99.8 %) solution. All subsequent washing steps with ethanol were performed with the same solution. The NWs were washed three times with ethanol. The NWs were transferred to a new tube, suspended in 5 mL ethanol, and intensively ultrasonicated on a water bath for 15 min to ensure uniform dispersion.

APTES (100 µL) was added to the NWs suspension and the reaction was left to proceed in a sonication bath for 1 h at 40 °C. After the first sonication step, 10 µL NaOH (1 M in H_2_O) and 200 µL deionized water (MilliQ^®^, Millipore) were added to the suspension to promote the base catalysis reaction. The suspension was sonicated further for another hour.

The resulting NWs were precipitated magnetically (using the DynaMag™-2 magnet rack), and washed with absolute ethanol. After the first washing step they were transferred to a 1.5 mL Eppendorf^®^ tube and subsequently washed four more times.

Infrared spectroscopy by attenuated total reflectance (ATR) was used to characterize the coating of Fe NWs with APTES. Measurements were taken with the Thermo Scientific™ Nicolet™ 6700/8700 FT-IR.

The surface charges, **ζ** (zeta) potential, of the APTES-coated Fe NWs and the non-coated Fe NWs for comparison were measured in deionized (DI) water using a Zetasizer Nano ZS, He–Ne laser 633 nm (Malvern Instruments, Malvern, UK).

Three replicates were used for each of the two measurements.

*Transmission electron microscopy (TEM)* observations were performed. Samples were prepared by diluting a solution of nanowires and depositing a drop of the solution on a copper grid coated with a thin film of amorphous carbon and allowing the liquid to air dry at RT.

Images were acquired with a Titan G2 80-300 CT microscope from FEI Company.

### Labeling of NWs with pHrodo™ red

pHrodo™ Red, succinimidyl ester (P 36600) was purchased from molecular probes™ of Thermo Fisher Scientific.

The labeling was based on the amide formation reaction between the succinimidyl-activated carboxylic acid group of the pHrodo™ Red complex and the free amino groups on the surface of the aminosilane -coated NWs. A schematic drawing of the reaction is shown in Additional file 8. The NWs coated with APTES were dried at room temperature (RT) (23 °C) for 30 min to allow ethanol to evaporate after the last washing steps. They were then suspended in 490 µL sodium bicarbonate buffer (NaHCO_3_, pH 8.4) and 10 µL pHrodo™ Red NHS ester dye was added. Previously, 1 mg pHrodo™ Red N-hydroxysuccinimide (NHS) ester was dissolved in 150 µL DMSO to afford a stock solution of approximately 10.2 mM. The tube was covered with Al (aluminum) foil to ensure protection from light and put on a thermomixer. The reaction was left to proceed for 12 h at RT, while shaking at 900 rpm (revolutions per minute).

The NWs were subsequently washed five times with the NaHCO_3_ buffer and three times with absolute ethanol. They were then suspended in 1 mL ethanol and stored at -20 °C.

### Cell culture and subculture

Cells were grown in a 37 °C humidified incubator with 5 % carbon dioxide (CO_2_).

Trypsin–EDTA (0.25 % Trypsin/0.53 mM EDTA in HBSS) was purchased from ATCC (30-2101).

HCT 116 (ATCC CCL247) cells were grown in 25 cm^2^ culture flasks in McCoy’s medium (McCoy’s 5A 1× medium with l-glutamine purchased from Mediatech, Inc.) with 10 % fetal bovine serum (FBS), and 100 IU mL^−1^ penicillin/0.1 mg/mL streptomycin solution.

HeLa (ATCC^®^ CCL-2™) cells were grown in 75 cm^2^ culture flasks in Dulbecco’s Modified Eagle’s medium (DMEM 1x high glucose, GlutaMax, pyruvate, purchased from Gibco of Thermo Fisher Scientific) with 10 % fetal bovine serum (FBS), and 100 IU mL^−1^ penicillin/0.1 mg/mL streptomycin solution.

For sub-culturing cells, a dilution was made in order to seed 1 × 10^6^ HeLa cells in a 75 cm^2^ culture flask (total volume of 21 mL), and 0.5 × 10^6^ HCT 116 cells in a 25 cm^2^ culture flask (total volume of 7 mL).

### Cell seeding

The Invitrogen™ Countess™ Automated Cell Counter was used for counting the cells.

35 mm plastic bottom dishes were used for the imaging experiments with a total surface area of 9 cm^2^. The seeding density for both HeLa and HCT 116 cells was 1.5 × 10^5^ cells, and they were seeded 48 h in advance of the time-lapse experiments.

The aim was to reach a confluence of 1.2 × 10^6^ cells (90 %) at the end of the 24 h time-lapse experiments for the given surface area. Nunclon^®^ cell culture dishes (Sigma-Aldrich) were used for the imaging experiments.

### Live cell imaging

Hoechst 33342 (Life technologies) was purchased from life technologies of Thermo Fisher Scientific.

The time-resolved cellular uptake studies were performed with the Nikon Biostation IM-Q CELL-S2-P model.

All time-lapse experiments were recorded at a resolution of 800 × 600 binning (recording pixels) with a 10× magnification. The total imaging time was 24 h with a time interval of 10 min between frames.

Shortly before the start of the time-lapse experiment, cells were washed three times with PBS (phosphate buffered saline, pH 7.4), stained with 10 µM Hoechst 33342 solution (Life technologies) for 15 min and subsequently rinsed with PBS three additional times. Images were obtained from the fluorescence emitted by pHrodo and Hoechst 33342.

The DAPI (4′,6-diamidino-2-phenylindole) and G-2A filters were used for imaging, with the following excitation filter (EX)/dichroic mirror (DM)/barrier filters (BA) characteristics: 350/400/460 for the Hoechst 33342 channel and 535/575/590 for the pHrodo™ Red channel. For the DAPI filter, an exposure time of 1/40 s was used (Epifluorescence lamp intensity 3 %), while for the G-2A filter, an exposure time of 1/4 s (Epifluorescence lamp intensity 12 %). For the transmission channel, a DIA lamp with exposure time of 1/40 s was used.

In order to avoid high background fluorescence we used Gibco^®^ FluoroBrite™ DMEM medium, without phenol red and riboflavin.

Prior to the time-lapse experiment, the Gibco^®^ FluoroBrite™ DMEM imaging media was pre-warmed to 37 *°*C before replacing the standard medium in the plastic bottom dish, to avoid mitotic entry delays.

At this point, NWs were added to the culture to interact with the cells. Different positions within the dish were defined for the time-lapse analysis. There was a delay of approximately 10 min between the addition of NWs and the start of the experiment. This was taken into account in the later quantitative time-resolved analysis.

### Imaging with Zeiss LSM 710, Axio Imager, upright confocal microscope

The cells were incubated with a 10 µM Hoechst 33342 solution for 15 min. After three washing steps with PBS, the cells were maintained at RT in extracellular buffer (ECB). The buffer solution contained (in mM): 125 NaCl, 26 NaHCO_3_, 20 glucose, 3 KCl, 1 NaH_2_PO_4_, 2 CaCl_2_ and 1 MgCl_2_, pH 7.4 when bubbled with a gas mixture consisting of 95 % O_2_ and 5 % CO_2_.

The emission of the pHrodo™ Red was detected with the TRITC (tetramethylrhodamine isothiocyanate) filter set with a confocal gain of 700 V of the photomultiplier tube (PMT). The water-immersion W Plan-Apochromat 63×/1.0 Ph3 M27 objective was used. The laser excitations were of 405 nm for Hoechst 33342 signal and of 561 nm for pHrodo™ Red signal.

### Flow cytometry analysis

Analysis was done on a BD LSRFortessa™Flow Cytometer (BD Biosciences). Fluorescence emission of pHrodo™ Red. pHrodo fluorescence was recorded after excitation with a 561 nm laser using a 610/20 nm filter (PE-Texas Red fluorescence). Data analysis was based on a total number of 50,000 harvested cells (single experiment) and performed using FlowJo software 7.6.1 (TreeStar Inc., Ashland, USA). Cells were seeded and grown in same conditions as described in the sections above.

### Image analysis with CellCognition

The CellCognition installation instructions are given in Additional file 9.

**Fig. 4:**
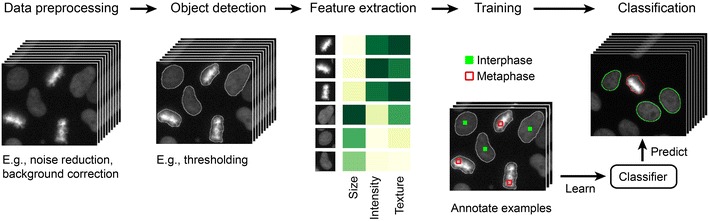
The machine learning pipeline for analysis of microscopy data. Reproduced with permission from [51]

The general steps for the analysis with CecogAnalyzer—the graphical user interphase of CellCogniton—are described in detail by Sommer and Gerlich and Held et al. [51, 52]. A schematic drawing of the machine learning pipeline is shown in Fig. 4.

The image pre-processing is aimed at clearing away artifacts produced by the microscope or camera [51]. In this step, typically, smoothing filters are used to remove pixel noise and cellular signal intensity levels are normalized by image flat-field correction [51].

The objects of interest, which form the basis for classification, are then detected by image segmentation using the object detection parameters (Additional file 10) [53]. Object detection is mainly based on pixel intensities, shape information and distance between objects. A nuclear marker (e.g. Hoechst) is generally used for the primary object detection, in order to distinguish individual cells.

Secondary object regions are derived for a secondary marker (fluorescent dye, e.g. pHrodo™ Red) on the basis of the primary segmentation marker. These secondary regions are expanded areas around the primary regions corresponding to the nuclear marker. The dimensions of these expanded areas are specified based on typical cell size and commonly include the entire cytoplasmic area.

The gray-value normalization is essential to object detection as it can exclude background/noise signal and ensure at the same time that no signal is lost. The lower range value (arbitrary units) used for the normalization is the value in the 16 bit image which corresponds to 0 in the 8 bit image, whereas the higher range value corresponds to 255 in the 8 bit image. These values are generally chosen based on fluorescence intensity measurements of the background and maximum intensity values for a few randomly chosen time-lapse images.

The third step of the pipeline, feature extraction, is performed for the objects of interest (cells) and the corresponding fluorescence channels. The main features extracted are size, circularity, geometry and texture. Advanced statistical features used for this step are described in the Additional file 11 [53].

The support vector machine classifier can be trained for discrimination of different object classes. The user defines these classes and manually annotates in the classification browser example objects for each class (e.g. cells with different characteristics). Examples are picked by visual observation of the object characteristics and are recorded with the set of features specified in the feature extraction step. For instance, cells in different phases of cell division can be distinguished by defining separate object classes and providing representative examples for each of the respective classes.

The machine learning algorithm is then trained on this example set to discriminate the different object classes. Cross-validation is performed on the training data set to ensure agreement between human and computer annotation.

Only upon ensuring that the machine learning algorithm inferred the rules to discriminate the classes (i.e. low cross-validation error), the full data set is analyzed.

For this study, CellCognition has been adapted to distinguish two classes of cells: cells with internalized NWs and cells with no internalized NWs. The settings, which have been used for the pipeline described in this study, are exhibited in Additional file 9.

**Fig. 5:**
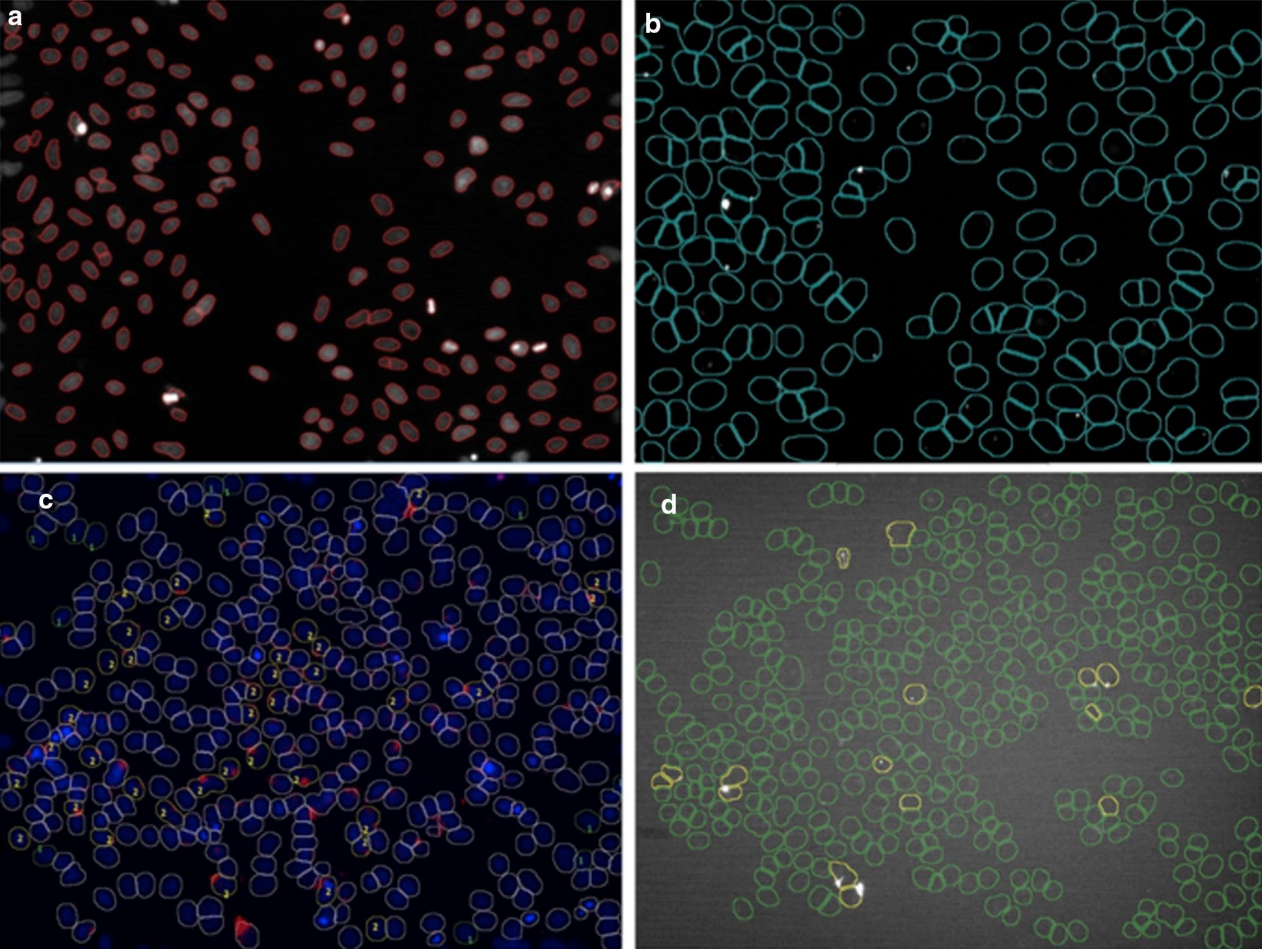
Screenshots of the main image analysis steps performed with CecogAnalyzer. **a** Object detection—primary channel. Object detection processing step for primary channel corresponding to Hoechst 33342 fluorescence. The contours in red correspond to the nuclear region of the cells, and define as such the number of cells per each time frame. **b** Object detection—secondary channel. Object detection processing step for secondary channel corresponding to pHrodo™Red fluorescence. The contours in *green* correspond to the area around the nucleus in which the pHrodo™Red signal (displayed in *white*) can be detected. **c** Manual annotation in the annotation browser. Examples picked for the two classes “nanonegative” and “nanopositive.” Hoechst 33342 fluorescence in *blue*, pHrodo™Red signal in *red*. “Nanopositive” cells indicated by “2”, “nanonegative” cells by “1.” **d** Automatic classification of cells approximately 3 h post-incubation with NWs. “Nanopositive” cells indicated by the *yellow* contour. “Nanonegative” cells indicated by the *green* contour

In our case, the Hoechst 33342 nuclear stain was used as a reference marker for image segmentation and the primary object detection step. A screenshot of the primary object detection step and the corresponding example is shown in Fig. 5a. The red contours define individual cells and mark their nuclear regions.

The secondary object region in this study was defined by an expanded area around the primary region corresponding to the Hoechst 33342 nuclear marker. A screenshot of the object detection for the secondary pHrodo™ Red channel and the corresponding example can be observed in Fig. 5b. The dimensions of this area were specified based on the typical cell size, and secondary object detection was based on the signal from the pHrodo™ Red channel in this expanded area. This signal is displayed in white in Fig. 5b and is found inside the green contours corresponding to the secondary object regions.

It should be noted that for HeLa cells, which are larger than HCT 116 cells, the expansion size was increased. A detailed description of the parameters used for object detection is found in the 4 [53].

For the gray-value normalization of the pHrodo™ Red channel, the range 100–800 was chosen (Min: 100; Max: 800) based on fluorescence intensity measurements of the background and maximum intensity values for a few arbitrarily chosen time-lapse images, using Image J.

By using the respective minimum value of 100, it was ensured that specific signal originating from well-defined spots in the pHrodo™ Red channel was not lost. However, there was a significant decrease in the background noise. As the software requires input from the user for initial annotation, such background noise can affect this process and the overall accuracy of the analysis. However, in the case of the experiment with HCT 116 cells and Ni NWs the background noise was larger than in the other conditions. A minimum value of 200 was used in this case instead of 100. It is our recommendation that for each particular experiment and condition, a different normalization should be performed based on the measurements of the background intensity for few arbitrarily chosen time-lapse images. We also recommend using FACS at early time points after incubation with nanostructures (3 and 6 h) for “validation” of the chosen fluorescence intensity normalization window. The choice of the minimum value in the gray-value normalization step is a critical step in the presented pipeline.

The maximum value of 800 was used for all experiments, following fluorescence intensity measurements of the brightest spots in the pHrodo™ Red channel for a few arbitrarily chosen time-lapse images.

The gray-value normalization for the pHrodo™ Red channel ensured that low intensity signal potentially originating from pHrodo™ Red-tagged NWs bound to the surface of the cell will be discriminated, thus addressing limitations of this dye previously remarked in literature [27].

For the feature extraction step, the basic shape features and convex hull features were not used in the case of the secondary pHrodo™ Red channel; gathering more features does not necessarily improve performance and makes the classification exponentially more complex [51].

Classification is central to machine learning and is the key step in the pipeline presented in this study.

For this project, the support vector machine classifier was trained for the discrimination of two different object classes—“nanopositive” (cells with internalized NWs) and “nanonegative” (cells with no internalized NWs).

These classes by manual annotation of approximately 20–50 example objects for each class, as shown in Fig. 5c. The “nanonegative” class was delimited in green and marked with label “1”, and the “nanopositive” class in yellow with label “2”.

As shown in Fig. 5c, the examples were picked by visual observation of the object characteristics. The “nanopositive” class is defined by the red fluorescence signal associated with the blue nuclear reference marker and found in the respective expanded region, while the “nanonegative” class is defined by the absence of such signal.

Cross-validation was performed on the training data set and additional examples were picked to ensure an agreement between human and computer annotation of at least 97 % accuracy.

Upon ensuring that the machine learning algorithm inferred the rules to discriminate the classes, the full data set was analyzed. Each full data set consisted of 145 image frames corresponding to 145 different time points within a 24 h time interval.

Figure 5d shows an example classification performed automatically by the program for an image frame corresponding to approximately 3 h post-incubation with NWs. The “nanopositive” cells are displayed in yellow and the “nanonegative” cells in green.

The object counts for the two classes previously defined, representing the number of “nanopositive” and “nanonegative” cells, were output by the software in a.txt file, for each time point of the 24 h time-lapse. The respective object counts allowed for a quantification of the number of cells with internalized NWs. Different areas with distinct populations of cells within the same cell dish were used for the analysis. The percentages of “nanopositive” cells for each area were calculated, averaged and reported with standard deviation.

## Additional files

10.1186/s12951-015-0153-x FTIR spectrum of Fe NWs. FTIR spectrum of non-coated Fe NWs and APTES-coated Fe NWs.
10.1186/s12951-015-0153-x FTIR spectrum of Ni NWs. FTIR spectrum of non-coated Ni NWs and APTES-coated Ni NWs.
10.1186/s12951-015-0153-x TEM characterization of APTES-coated Fe NW.
10.1186/s12951-015-0153-x TEM characterization of APTES-coated Ni NW.
10.1186/s12951-015-0153-x Time-lapse imaging of HeLa cells with pHrodo™ Red-tagged Ni NWs. Video showing large NW aggregates torn down by cells. The duration of the video corresponds to a period of 24 h.
10.1186/s12951-015-0153-x Time-lapse imaging of HeLa cells with pHrodo™ Red-tagged Fe NWs. Video showing cells, which are dividing, with NW aggregates. The duration of the video corresponds to a period of 24 h.
10.1186/s12951-015-0153-x Control experiment with iron nanowires and cell imaging medium adjusted for different pH values. A) pH 8.2 – measured value for Fluorobrite imaging medium. B) pH 6.3 – value corresponding to pH inside early endosomal vesicles.
10.1186/s12951-015-0153-x Fe NWs coating with APTES and labeling with pHrodo red. Fe NWs were coated with APTES initially and subsequently labeled with pHrodo red based on the reaction between the succinimidyl ester group of the pHrodo red complex and the surface amino groups of the APTES-coated Fe NWs.
10.1186/s12951-015-0153-x Settings used for analysis with CellCognition.
10.1186/s12951-015-0153-x Object detection parameters.
10.1186/s12951-015-0153-x Feature types available in CecogAnalyzer.

## Abbreviations

Al: aluminum
APTES: 3-Aminopropyl triethoxysilane
ATR: attenuated total reflectance
BA: barrier filters
DI: deionized
DM: dichroic mirror
DMEM: Dulbecco’s Modified Eagle Medium
ECB: extracellular buffer
EX: excitation filter
FACS: fluorescence activated cell sorting
FBS: fetal bovine serum
Fe: iron
FTIR: Fourier transform infrared spectroscopy
HUVEC: Human vascular endothelial cells
NA: numerical aperture
NHS: *N*-hydroxysuccinimide esters
Ni: Nickel
NP: nanoparticle
NW: nanowire
PAO: porous aluminum oxide
PBS: phosphate buffered saline
PMT: photomultiplier tube
RPM: revolutions per minute
UCNP: upconversion nanoparticle
