# Supplementary figures and images for: Semi-automated quantification of living cells with internalized nanostructures
Source: J Nanobiotechnology. 2016 Jan 15;14:4. doi: 10.1186/s12951-015-0153-x (PMC4714438; doi:10.1186/s12951-015-0153-x)

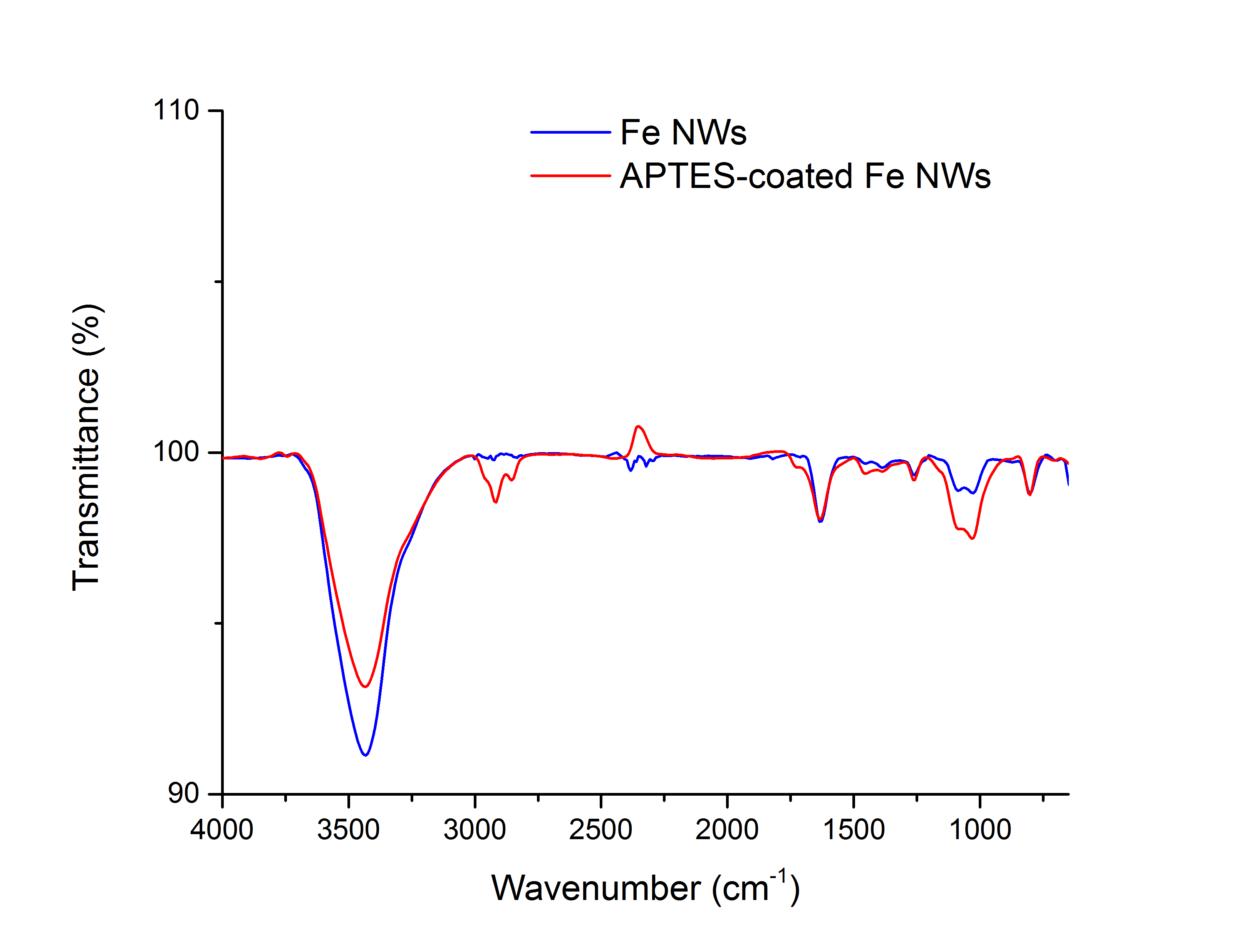

Supplement: Supplementary file 1 — 10.1186/s12951-015-0153-x FTIR spectrum of Fe NWs. FTIR spectrum of non-coated Fe NWs and APTES-coated Fe NWs. [file 12951_2015_153_MOESM1_ESM.png]

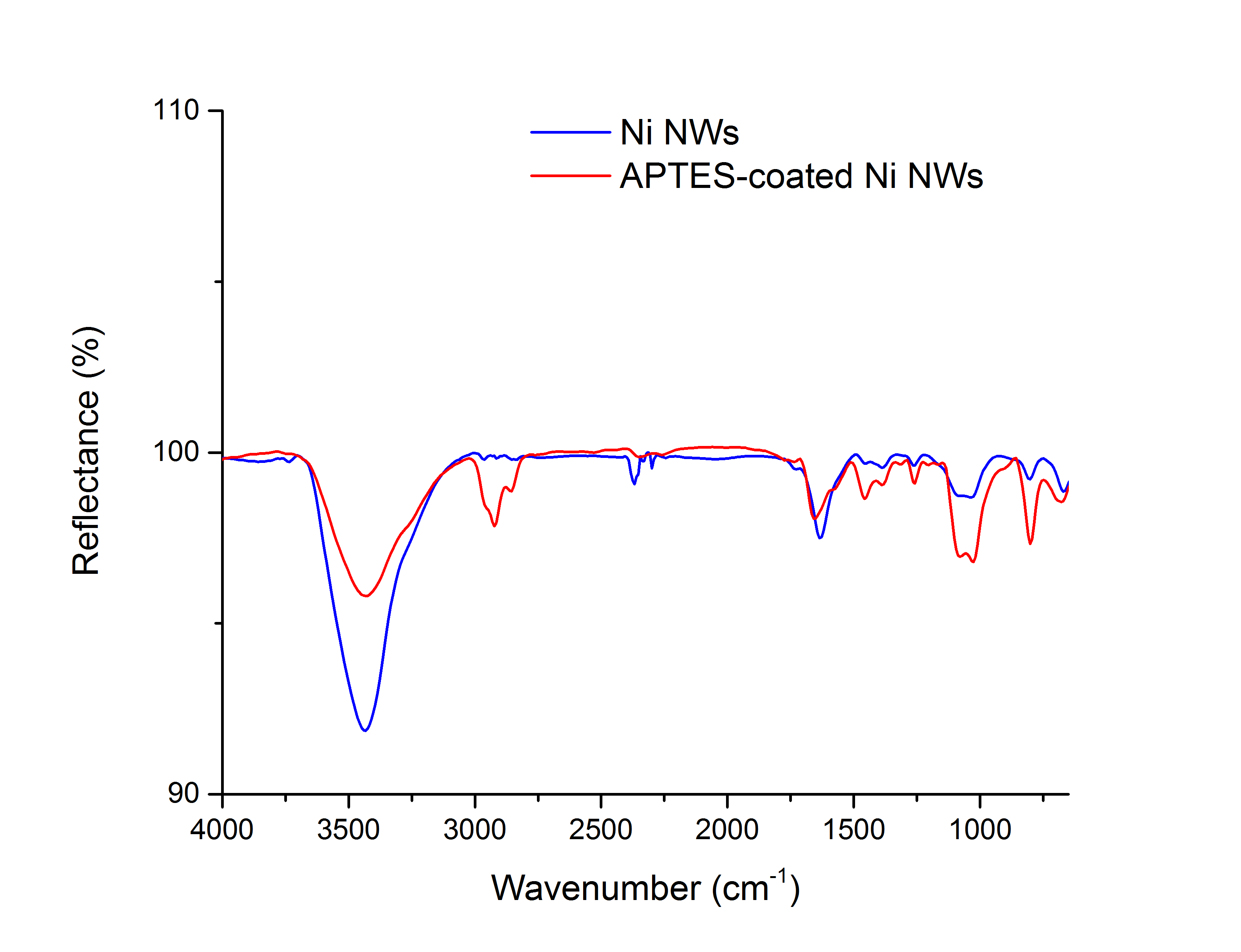

Supplement: Supplementary file 2 — 10.1186/s12951-015-0153-x FTIR spectrum of Ni NWs. FTIR spectrum of non-coated Ni NWs and APTES-coated Ni NWs. [file 12951_2015_153_MOESM2_ESM.png]

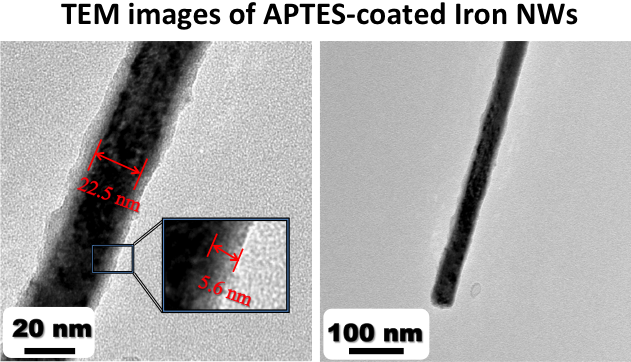

Supplement: Supplementary file 3 — 10.1186/s12951-015-0153-x TEM characterization of APTES-coated Fe NW. [file 12951_2015_153_MOESM3_ESM.png]

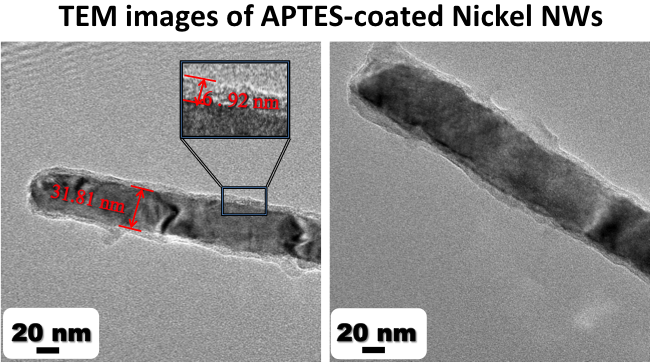

Supplement: Supplementary file 4 — 10.1186/s12951-015-0153-x TEM characterization of APTES-coated Ni NW. [file 12951_2015_153_MOESM4_ESM.png]

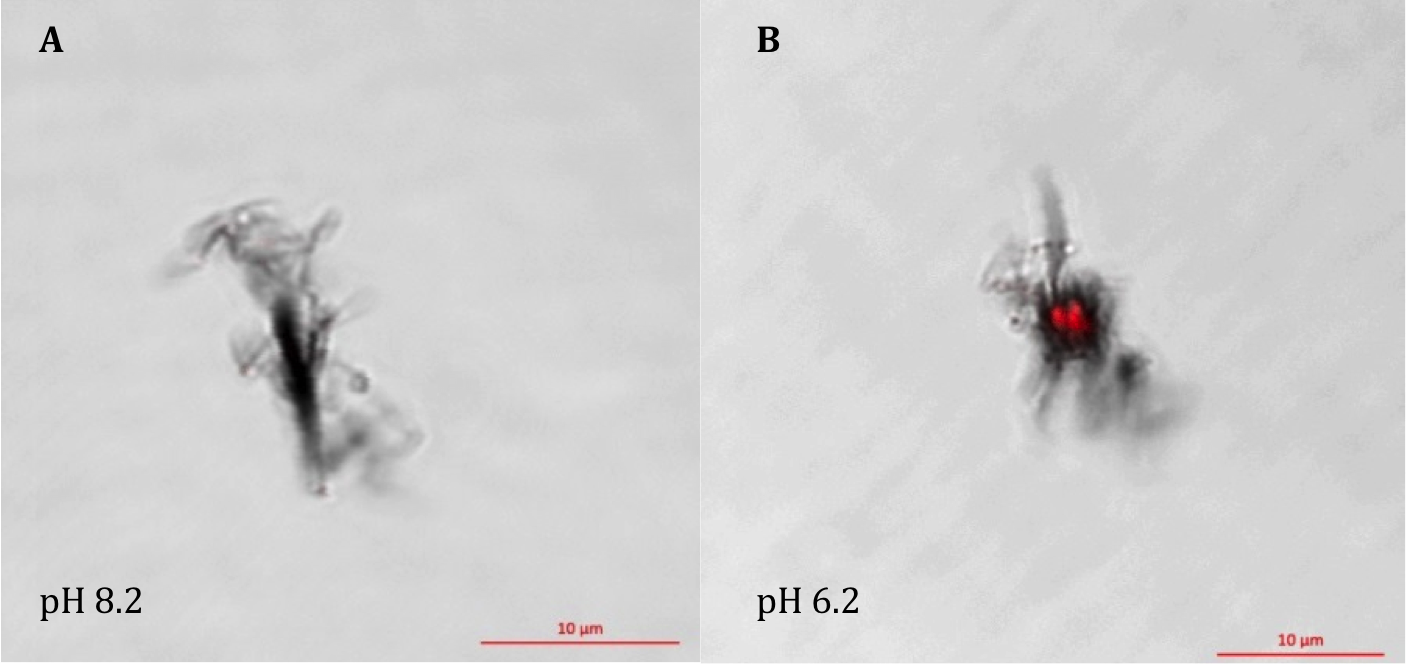

Supplement: Supplementary file 7 — 10.1186/s12951-015-0153-x Control experiment with iron nanowires and cell imaging medium adjusted for different pH values. A) pH 8.2 – measured value for Fluorobrite imaging medium. B) pH 6.3 – value corresponding to pH inside early endosomal vesicles. [file 12951_2015_153_MOESM7_ESM.png]

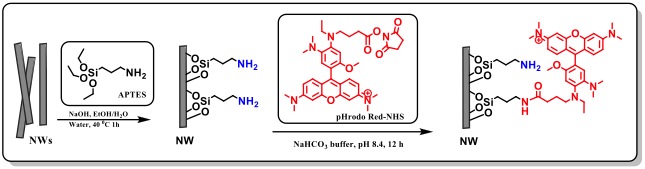

Supplement: Supplementary file 8 — 10.1186/s12951-015-0153-x Fe NWs coating with APTES and labeling with pHrodo red. Fe NWs were coated with APTES initially and subsequently labeled with pHrodo red based on the reaction between the succinimidyl ester group of the pHrodo red complex and the surface amino groups of the APTES-coated Fe NWs. [file 12951_2015_153_MOESM8_ESM.jpg]
