# Supplementary material for: Semi-automated quantification of living cells with internalized nanostructures
Source: J Nanobiotechnology. 2016 Jan 15;14:4. doi: 10.1186/s12951-015-0153-x (PMC4714438; doi:10.1186/s12951-015-0153-x)
Supplement: Supplementary file 9 — 10.1186/s12951-015-0153-x Settings used for analysis with CellCognition. [file 12951_2015_153_MOESM9_ESM.docx]

##The Cecog Analyzer software was downloaded from: http://gerlichlab.imba.oeaw.ac.at/software/cellcognition/CecogAnalyzer_1.5.2_x86_64.exe

##In the installation folder (e.g. C:\Program Files\CecogAnalyzer1.5.2), "resources" subfolder, the file "naming_schemas.ini" was edited with the following section which was added after the last naming scheme “[EMBL_ScanR_well_subfolder]”:

[Nanostructures]

file_extensions = .tiff .tif

regex_subdirectories = ^[^_].*

regex_filename_substr = (.+?)\.

regex_dimensions = (?P<channel>[A-Za z\d]+)_P(?P<well>\d+)_Z(?P<zslice>\d+)_S(?P<subwell>\d+)_T(?P<time>\d+)

timestamps_from_file = mtime

## The settings adapted for the study presented here, which were loaded in the General tab of the software, are shown below:

[General]

image_import_namingschema = True

crop_image_y1 = 600

crop_image_y0 = 0

framerange_end = 10

constrain_positions = False

primary_featureextraction_exportfeaturenames = ['n2_avg', 'n2_stddev', 'roisize']

pathout = C:\…\Analysis

structure_file_pathout = False

structure_filename =

structure_file_extra_path = False

framerange_begin = 0

has_multiple_plates = False

pathin = C:\…\Images

framerange = False

structure_file_pathin = True

image_import_structurefile = False

tertiary_featureextraction_exportfeaturenames = ['n2_avg', 'n2_stddev', 'roisize']

crop_image_x0 = 0

crop_image_x1 = 800

namingscheme = Nanostructures

crop_image = False

secondary_featureextraction_exportfeaturenames = ['n2_avg', 'n2_stddev', 'roisize']

positions = 0037

redofailedonly = False

frameincrement = 1

structure_file_extra_path_name =

version = 1.5.2

process_merged = False

process_tertiary = False

process_primary = True

process_secondary = False

rendering = {'primary_contours_primary': {'Primary': {'raw': ('#FFFFFF', 1.0), 'contours': [('primary', '#FF0000', 1, False)]}}}

rendering_class = {'primary_classification_primary': {'Primary': {'raw': ('#FFFFFF', 1.0), 'contours': [('primary', 'class_label', 1, False), ('primary', '#000000', 1, False)]}}}

[ObjectDetection]

secondary_channelid = pHrodo

tertiary_normalizemax = 10000

tertiary_zslice_projection = False

tertiary_channelregistration_x = 0

tertiary_normalizemin = 0

primary_zslice_selection_slice = 1

primary_zslice_selection = True

primary_zslice_projection_end = 1

secondary_zslice_projection = False

tertiary_zslice_projection_begin = 1

tertiary_zslice_projection_method = maximum

primary_zslice_projection = False

tertiary_channelid = Phase

primary_channelid = Hoechst

primary_zslice_projection_begin = 1

tertiary_channelregistration_y = 0

primary_normalizemax = 2500

secondary_zslice_selection = True

primary_normalizemin = 0

primary_zslice_projection_step = 1

primary_flat_field_correction = False

secondary_normalizemax = 800

secondary_normalizemin = 100

secondary_channelregistration_y = 0

secondary_channelregistration_x = 0

tertiary_flat_field_correction = False

tertiary_zslice_selection_slice = 1

secondary_zslice_selection_slice = 1

tertiary_zslice_projection_step = 1

tertiary_zslice_projection_end = 1

secondary_flat_field_correction = False

secondary_zslice_projection_begin = 1

tertiary_zslice_selection = True

secondary_zslice_projection_method = maximum

primary_zslice_projection_method = maximum

secondary_zslice_projection_step = 1

secondary_zslice_projection_end = 1

plugin__primary_segmentation__primary__primary__lat2 = False

plugin__primary_segmentation__primary__primary__intensitywatershed = False

plugin__primary_segmentation__primary__primary__intensitywatershed_gausssize = 5

plugin__primary_segmentation__primary__primary__postprocessing_intensity_max = -1

plugin__primary_segmentation__primary__primary__postprocessing_intensity_min = -1

plugin__primary_segmentation__primary__primary__latwindowsize = 17

plugin__primary_segmentation__primary__primary__postprocessing_roisize_min = 16

plugin__primary_segmentation__primary__primary__removeborderobjects = True

plugin__primary_segmentation__primary__primary__holefilling = True

plugin__primary_segmentation__primary__primary__postprocessing_roisize_max = -1

plugin__primary_segmentation__primary__primary__latlimit2 = 5

plugin__primary_segmentation__primary__primary__postprocessing = True

plugin__primary_segmentation__primary__primary__shapewatershed_gausssize = 2

plugin__primary_segmentation__primary__primary__intensitywatershed_maximasize = 11

plugin__primary_segmentation__primary__primary__shapewatershed_minmergesize = 30

plugin__primary_segmentation__primary__primary__medianradius = 2

plugin__primary_segmentation__primary__primary__intensitywatershed_minmergesize = 95

plugin__primary_segmentation__primary__primary__latwindowsize2 = 22

plugin__primary_segmentation__primary__primary__shapewatershed_maximasize = 9

plugin__primary_segmentation__primary__primary__shapewatershed = True

plugin__primary_segmentation__primary__primary__latlimit = 3

merged_zslice_selection = True

primary_channelregistration_y = 0

primary_channelregistration_x = 0

merged_channelid =

merged_zslice_projection = False

merged_channelregistration_x = 0

merged_normalizemin = 0

merged_zslice_projection_method = maximum

merged_normalizemax = 255

merged_zslice_projection_begin = 1

merged_zslice_projection_step = 1

primary_flat_field_correction_image_dir =

merged_flat_field_correction_image_dir =

secondary_flat_field_correction_image_dir =

merged_zslice_projection_end = 1

merged_zslice_selection_slice = 1

merged_channelregistration_y = 0

tertiary_flat_field_correction_image_dir =

merged_flat_field_correction = False

plugin__secondary_segmentation__expanded__expanded__expansion_size = 3

plugin__secondary_segmentation__expanded__expanded__require00 = primary

plugin__secondary_segmentation__outside__outside__expansion_size = 5

plugin__secondary_segmentation__outside__outside__separation_size = 1

plugin__secondary_segmentation__outside__outside__require00 = primary

[FeatureExtraction]

primary_featurecategory_stat_geom = True

primary_featurecategory_granugrey = True

tertiary_featurecategory_intensity = True

primary_featurecategory_basicshape = True

primary_featurecategory_convhull = True

primary_featurecategory_moments = True

secondary_featurecategory_basicshape = True

secondary_featurecategory_granugrey = True

secondary_featurecategory_stat_geom = True

tertiary_featurecategory_haralick = True

secondary_featurecategory_convhull = True

primary_featurecategory_intensity = True

secondary_featurecategory_distance = True

tertiary_featurecategory_moments = True

secondary_featurecategory_intensity = True

tertiary_featurecategory_basicshape = True

primary_featurecategory_distance = True

tertiary_featurecategory_stat_geom = True

secondary_featurecategory_haralick = True

tertiary_featurecategory_granugrey = True

primary_featurecategory_haralick = True

tertiary_featurecategory_convhull = True

tertiary_featurecategory_distance = True

secondary_featurecategory_moments = True

[Classification]

secondary_classification_envpath = c:\…\Application Data\CellCognition1.5.2\battery_package/Classifier/aTubulin

collectsamples = False

secondary_classification_regionname = expanded

tertiary_classification_envpath =

primary_classification_regionname = primary

primary_classification_envpath = c:\…\Application Data\CellCognition1.5.2\battery_package/Classifier/H2B

tertiary_classification_regionname =

collectsamples_prefix =

merge_primary = True

merged_classification_envpath =

merge_secondary = True

merge_tertiary = False

merged_classification_regionname =

merged_secondary_region = expanded

merged_primary_region = primary

merged_tertiary_region =

[Tracking]

tracking_maxtrackinggap = 5

tracking_centroid_radius = 3

tracking_maxobjectdistance = 36

tracking_compressiontrackfeatures = raw

tracking_visualize_track_length = -1

tracking_visualization = True

tracking_maxsplitobjects = 2

tracking_exporttrackfeatures = True

region = primary

[ErrorCorrection]

groupby_position = False

constrain_graph = False

mappingfile_path =

timelapse = 4.5

ignore_tracking_branches = False

primary_graph = c:\…\Application Data\CellCognition1.5.2\battery_package/Settings/graph_primary.xml

secondary_graph = c:\…\Application Data\CellCognition1.5.2\battery_package/Settings/graph_secondary.xml

sorting_sequence =

secondary_sort = NULL

groupby_oligoid = True

compose_galleries_sample = -1

groupby_genesymbol = False

overwrite_time_lapse = False

primary_sort = NULL

max_time = 100.0

enable_sorting = False

position_labels = False

compose_galleries = False

primary = True

tertiary = False

merged_graph =

tertiary_graph =

secondary = False

merged = False

resampling_factor = 0.4

hmm_baumwelch = False

hmm_smoothing = True

size_gallery_image = 60

[Output]

rendering_labels_discwrite = False

events_export_gallery_images = False

hdf5_include_raw_images = False

rendering_class_showids = False

export_object_details = False

export_object_counts = False

rendering_class_discwrite = False

hdf5_include_features = True

events_export_all_features = False

export_tracking_as_dot = False

events_gallery_image_size = 50

hdf5_create_file = True

rendering_contours_showids = False

rendering_contours_discwrite = False

export_track_data = False

hdf5_include_label_images = False

export_object_counts_ylim_max = -1

rendering_channel_gallery = False

export_file_names = False

hdf5_include_events = True

hdf5_merge_positions = True

hdf5_include_tracking = True

hdf5_include_crack = True

hdf5_compression = True

hdf5_include_classification = True

hdf5_reuse = False

export_events = False

[Processing]

tertiary_featureextraction = False

secondary_featureextraction = True

tracking = True

secondary_errorcorrection = False

primary_errorcorrection = False

tertiary_errorcorrection = False

primary_classification = True

objectdetection = True

tertiary_classification = False

secondary_classification = True

primary_featureextraction = True

merged_classification = False

merged_featureextraction = False

merged_errorcorrection = False

eventselection = False

[Cluster]

position_granularity = 1

[EventSelection]

forwardrange = 5.0

forwardrange_min = False

maxindegree = 1

labeltransitions = (2,3)

event_selection = True

forwardcheck = 2.0

supervised_event_selection = True

forwardlabels = 3,4,5,6,7,8,9

backwardrange_min = False

backwardrange = 5.0

duration_unit = frames

maxoutdegree = 2

backwardcheck = 2.0

min_event_duration = 3

min_cluster_size = 2

unsupervised_event_selection = False

num_clusters = 6

backwardlabels = 1,2

eventchannel = Primary

[PostProcessing]

color_sort_by_position = False

group_by_oligoid = False

mappingfile_path =

single_plot_max_plots = 1

group_by_position = True

single_plot_ylim_low = 1.0

group_by_genesymbol = False

nebd_onset_factor_threshold = 1.2

ibb_onset_factor_threshold = 1.2

color_sort_by_genesymbol = False

single_plot_ylim_high = 5

color_sort_by_group = False

securin_analysis = True

single_plot = True

group_by_group = False

ibb_range_signal_threshold = 3.0

ibb_analysis = True

color_sort_by_oligoid = True

plot_ylim1_high = 100

ibb_ratio_signal_threshold = 1.2

plot_ylim1_low = 0
